# Joint triplet loss with semi-hard constraint for data augmentation and disease prediction using gene expression data

**DOI:** 10.1038/s41598-023-45467-8

**Published:** 2023-10-24

**Authors:** Yeonwoo Chung, Hyunju Lee

**Affiliations:** 1 School of Electrical Engineering and Computer Science, Gwangju Institute of Science and Technology, Gwangju, 61005 Republic of Korea; 2 Artificial Intelligence Graduate School, Gwangju Institute of Science and Technology, Gwangju, 61005 Republic of Korea

**Keywords:** Cancer, Computational models

## Abstract

The accurate prediction of patients with complex diseases, such as Alzheimer’s disease (AD), as well as disease stages, including early- and late-stage cancer, is challenging owing to substantial variability among patients and limited availability of clinical data. Deep metric learning has emerged as a promising approach for addressing these challenges by improving data representation. In this study, we propose a joint triplet loss model with a semi-hard constraint (JTSC) to represent data in a small number of samples. JTSC strictly selects semi-hard samples by switching anchors and positive samples during the learning process in triplet embedding and combines a triplet loss function with an angular loss function. Our results indicate that JTSC significantly improves the number of appropriately represented samples during training when applied to the gene expression data of AD and to cancer stage prediction tasks. Furthermore, we demonstrate that using an embedding vector from JTSC as an input to the classifiers for AD and cancer stage prediction significantly improves classification performance by extracting more accurate features. In conclusion, we show that feature embedding through JTSC can aid in classification when there are a small number of samples compared to a larger number of features.

## Introduction

Deep learning methods have improved prediction accuracy for a wide range of tasks in the biomedical field^1–3^. However, in most cases, the amount of biomedical data is small owing to the high cost of data collection. Thus, several computational efforts have been made to overcome this lack of data. In the case of image data, data augmentation methods have been used to prevent overfitting and train more complex models^4,5^. Generative adversarial networks (GAN) is a leading data augmentation technique based on the zero-sum principle^6^. GAN trains two neural networks, a generator, and a discriminator. While the discriminator distinguishes the generated data from the real data, the generator strives to generate synthetic data that closely adheres to the original data distribution. Recently, deep metric learning with triplet loss has been proposed to overcome the limitations of small datasets. Deep metric learning is an approach for learning metric embedding^7^. It learns the representation of the input data in a low-dimensional vector space by calculating the distance between vectors. Embeddings have been trained to obtain similar representations for the same class of data through the model. Recently, many loss functions have been developed for deep metric learning, such as contrastive, triplet, and quadruplet losses^8,9^. The Euclidean distance and cosine similarity are widely used as distance functions to bring samples of the same class closer together and others further apart. A triplet network passes three instances of anchor, positive, and negative samples^10^. It calculates pairs of positive and negative distances from the anchor and gives an advantage to training by generating a large triplet structure from a relatively small amount of data.

Chaudhari et al.^11^ and Viñas et al.^12^ proposed GAN-based data augmentation studies for gene expression data. Moreover, Moreno-Barea et al.^13^ developed a conditional GAN method using gene expression data, and Ahmen et al.^14^ proposed a GAN architecture that integrates two omics datasets to generate omics data from the other omics dataset. However, compared to GAN, deep metric learning has been less applied to gene expression data.

In the present study, we developed a new deep metric learning based model and applied it to gene expression profiles from Alzheimer’s disease (AD) and The Cancer Genome Atlas (TCGA) datasets. AD is a complex disease that causes memory loss. The number of patients with AD has increased in recent decades with the increase in life expectancy^15^. Accumulation of plaques (beta-amyloid) and tangles (tau) are generally identified as causes of the disease^16^, but the detailed pathogenic mechanisms are unknown. In the characterization of gene expression values in patients with AD, several studies have identified AD-related genes and discovered other potential candidates^17,18^. However, these studies used gene expression in brain tissues, which is invasive and cannot be used for the early diagnosis of AD. However, in the blood, there are differentially expressed proteins similar to those found in the brains of patients with AD^19^. Therefore, gene expression and protein levels in the blood have been investigated for the early diagnosis of AD^20,21^.

With regard to cancer, several studies have predicted patient survival and identified biomarkers for predicting cancer type and biological changes based on gene expression^22–24^. Aouiche et al.^25^ used a pathway network to extract stage-specific genes by constructing gene modules. Park et al.^26^ applied deep learning to stage prediction in gene expression profiles, and Rahimi et al.^27^ improved the performance of cancer stage prediction and identified gene sets that are commonly related across different cancer cohorts in TCGA. Kwon et al.^28^ applied GAN to data augmentation to address the problem of the small number of clinical samples and increase prediction accuracy.

As convolutional neural networks (CNN) are used in numerous applications, simplified CNN as 1D-CNN have been proposed for use in modeling 1-dimensional features^29^. It can better capture features from unbalanced data with a larger number of features than from the number of samples in biomedical data classification. The model detects global features with a minimal kernel stride by making the CNN stride equal to the kernel size^30^. Therefore, the 1D-CNN model was used as the embedding layer in our experiments using gene expression data.

We developed a new approach, called joint triplet loss with semi-hard constraint (JTSC), to mine more accurate triplets and identify improved performance by overcoming the lack of data. First, we trained a 1D-CNN model with a vector representation by generating sufficient triplets for training and obtained a classifier with improved prediction performance using these embedded vectors. Second, we propose a semi-hard constraint for sophisticated semi-hard mining and joint loss for training the vector representations. Finally, while learning the embedding function, we analyzed the change in the number of triplet categories to check whether the training of the embedding function was performed properly. When we measured the prediction performance of the proposed method using AD and cancer datasets from TCGA, the performance was improved by overcoming the lack of data.

## Methods

### Data description

We used peripheral blood gene expression profiles, such as GSE63060 (AddNeuroMed1, ANM1) and GSE63061 (AddNeuroMed2, ANM2), downloaded from the Gene Expression Omnibus (GEO) (https://www.ncbi.nlm.nih.gov/geo/). These expression profiles were generated using the Illumina HumanHT-12 v3.0 Expression BeadChip for the ANM1, and Illumina HumanHT-12 v4.0 Expression BeadChip for the ANM2. The other peripheral blood AD dataset was downloaded from the Alzheimer’s Disease Neuroimaging Initiative (ADNI) (http://adni.loni.usc.edu). We used all samples in ANM1, ANM2, and ADNI; however, samples without clinical information were excluded. We converted the probe ID to the Entrez Gene ID with the information from GPL6947 and GPL10558 for ANM1 and ANM2, respectively. Probe IDs not assigned to Entrez gene IDs were removed. We selected protein-coding genes in the assigned Entrez Gene ID based on the Homo_sapiens.GRCh38.94 database (http://asia.ensembl.org/Homo_sapiens/Info/Index); Ensembl IDs of protein-coding genes in the database were converted into Entrez Gene IDs using the “biomaRt” package in R software. The expression values for the duplicated Entrez gene IDs in protein-coding genes were treated as mean values. Subsequently, 16,730, 14,957, and 20,384 gene expression values were selected from ANM1, ANM2, and ADNI, respectively.

In AD, age and sex are prominent risk factors for dementia and are key features used for AD prediction^31^. Therefore, we normalized each clinical information and gene expression dataset and concatenated these normalized features. Samples without clinical information in each dataset were excluded from the prediction. In addition, age and sex information were used for ANM data analysis, and educational and marital information were additionally treated in the ADNI dataset (Supplementary Table S1). Marriage information was converted from − 2 to 2 based on marital status and 0 for unknown. Ten-fold cross-validation was performed, and 20 percent of the training set was allocated to the validation set.

We further tested the performance of our method by separating patients with early- and late-stage cancer for 14 cancers, namely, BRCA, COAD, ESCA, HNSC, KICH, KIRC, LIHC, LUAD, LUSC, PAAD, READ, STAD, TGCT, and THCA, in TCGA dataset. The data cohorts used are publicly available and were downloaded from the UCSC Xena Browser. Moreover, we obtained clinical information such as age, sex, and cancer stage from the GDC Data Portal (portal.gdc.cancer.gov), and the samples without clinical information were removed, identical to the data processing of the AD dataset. Note that we did not use the KIRP dataset, which does not include the cancer stage of the samples in the clinical information.

Among the cancer samples, primary tumor samples, not metastatic, were selected from each TCGA cohort. We considered stage I as early-stage and the remaining tumor stages as late-stage cancers^27^ (Supplementary Table S2). Protein-coding genes were selected using the Homo_sapiens.GRCh38.94 database for analysis. The duplicated official gene symbol was integrated into a unique gene symbol using the mean value of the gene expression profiles. As a result, we obtained the expression values of 16,561 protein-coding genes from the 20,530 genes for 13 cancers. For STAD, the expression values of 16,995 protein-coding genes among 26,540 genes were also treated using the same process. The characteristics of the samples in each stage are provided in Supplementary Table S3.

### 1D-CNN

**Figure 1:**
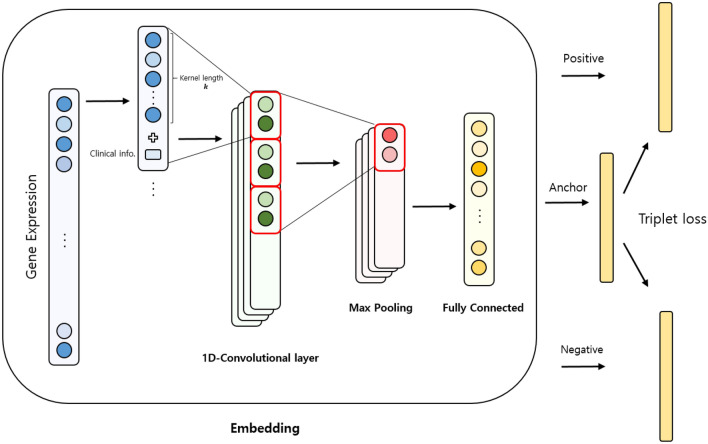
The pipeline of training triplet networks. Clinical information is concatenated with gene expression values before passing through the convolutional layer. Embedded vectors of the anchor, positive, and negative samples were trained such that samples from the same classes were calculated to be closer, whereas samples from a different class were calculated to be farther apart.

CNN structures are the most widely used in deep learning, especially in computer vision applications. A CNN that considers each surrounding pixel’s information can train a large amount of image data with a deep layer structure and many parameters. Based on the recent development of CNN, the 1D-CNN model is being utilized in computer vision and speech recognition^32,33^ as well as in various fields dealing with non-2-dimensional data. 1D-CNN also performed well in predicting cancers using TCGA data compared to other 2D-CNN structures^30^. In the 1D-CNN, the local information calculated by the convolutional kernel is also important. However, the previous study showed that it can perform well even when using randomly-ordered genes in the gene expression data^30^. When dealing with clinical information in the 1D-CNN model, we reconstructed datasets by concatenating the clinical information for each kernel size of gene expression to include the clinical information in the computation of the kernel unit for training (Fig. 1).

### Triplet loss and angular loss

Metric learning maps the data into a new vector space using an embedding function and helps a model train with better performance than when using the original features^34^. In general, metric learning uses Euclidean distance or cosine similarity as a distance metric between embedded data to learn the function from which the same class gets closer, and other classes move away. Deep metric learning uses a deep-layer model to learn these nonlinear embedding functions, and contrastive, triplet, and quadruplet embeddings are representative^9,35^. Triplet embedding consists of anchor, positive, and negative samples. Anchor *a* can belong to any class, positive samples *p* are extracted from the same class as the anchor, and negative samples *n* are extracted from a different class^36^. An embedding function $f : R^{d_{input}}\rightarrow R^{d_{embedding}}$ embeds the features of the input dimension $d_{input}$ into the low-dimensional space of the embedding dimension $d_{embedding}$ using the weight matrix $W \in R^{d_{input}*d_{embedding}}$. It then trains the vector of embedded anchor samples ($x_a = f(a)$) such that it becomes closer to the vector of embedded positive samples ($x_p = f(p)$). The embedding function *f* also trains the embedded vector of negative samples ($x_n = f(n)$) to be further away from the anchor. It minimizes the distance $d_p$ between the anchor and the positive sample and maximizes the distance $d_n$ between the anchor and the negative sample. In our experiments, the Euclidean distance was used to calculate distances $d_p$ and $d_n$. A margin *m* indicates the degree to which $d_n$ is judged to be close to $d_p$. The triplet loss with a hyperparameter margin *m* (> 0) is defined as follows:

$$\begin{aligned}{} & {} l_{triplet} = [d_p - d_n + m]_+,\\{} & {} l_{triplet} = [||x_a-x_p||^2 - ||x_a-x_n||^2 + m]_+ \end{aligned}$$

Moreover, there are three types of triplet samples: easy, semi-hard, and hard, depending on the relationship between $d_p$ and $d_n$. The easy sample is a sample in which the embedded vector of the positive sample is much closer to the anchor than the negative sample ($d_p$ $<< d_n$). The semi-hard sample is an easy sample, but it is harder than the easy sample because the difference between $d_p$ and $d_n$ is small ($d_n-d_p < m$). If the negative sample is closer to the anchor and the positive sample is farther from the anchor ($d_p$>>$d_n$), it is called a hard sample. In most cases of deep metric learning using triplet loss, semi-hard mining is used because including hard samples in the training process can lead to bad local optima^36^. Therefore, we constructed random *N* triplets out of all possible $n^3$ triplets in *n* data samples, and selected only semi-hard samples for training. We constructed a half of *N* triplets containing disease anchors and the other half of *N* triplets containing control anchors for balanced anchor training. An online semi-hard mining strategy was used to prevent poor training and converge quickly in each mini-batch. Unlike offline mining, which defines triplet categories from samples at the beginning of the epoch at once, we calculated distances from all *N* triplets and defined easy, semi-hard, and hard samples at each epoch to determine which semi-hard samples to use for weight updates.

An angular loss can achieve better similarity than the traditional triplet loss^37^. The angular loss compares the relative ratio of edges and treats all three edges, which is different from the traditional triplet loss, which deals with only two edges from $x_a$. When minimizing the angle ($\angle n$) in the negative sample, a triangle of three triplet points was constructed for stable training. The center point $x_c$ is defined as the average of the anchor point $x_a$ and positive point $x_p$ and hyperplane *P* is perpendicular to the edges of $x_c$ and $x_n$. Then, $x_m$ is defined as one of the intersection points between *P* and circle *C* with the edges of $x_a$ and $x_p$ as the diameter. A stable triplet triangle consists of $x_c$, $x_m$, and $x_n$ for training with an angular constraint. With the hyperparameter $\alpha$ as the degree of angular constraint ($\angle n \le \alpha$), it has an interpretable geometry, meaning that the angle constraint forms a skinny triangle that places negative samples away from the circle of the same class as the anchor. Therefore, an angular loss is a similarity transform based on the constraint that the angle is proportional to the relative ratio between the two distance differences.

$$\begin{aligned}{} & {} \angle n \le \alpha , \\{} & {} tan\angle n = {\frac{||x_m-x_c||}{||x_n-x_c||}} = {\frac{||x_a-x_p||}{2||x_n-x_c||}} \le tan\alpha ,\\{} & {} l_{angular} = [||x_a-x_p||^2-4tan^2\alpha ||x_n-x_c||^2]_+ \end{aligned}$$

### Semi-hard constraint under switching condition and loss function

**Figure 2:**
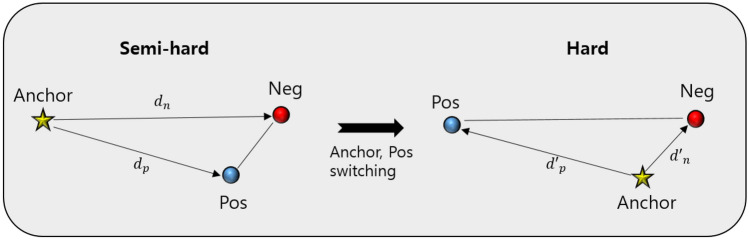
Category change of semi-hard triplet samples under switching condition.

**Figure 3:**
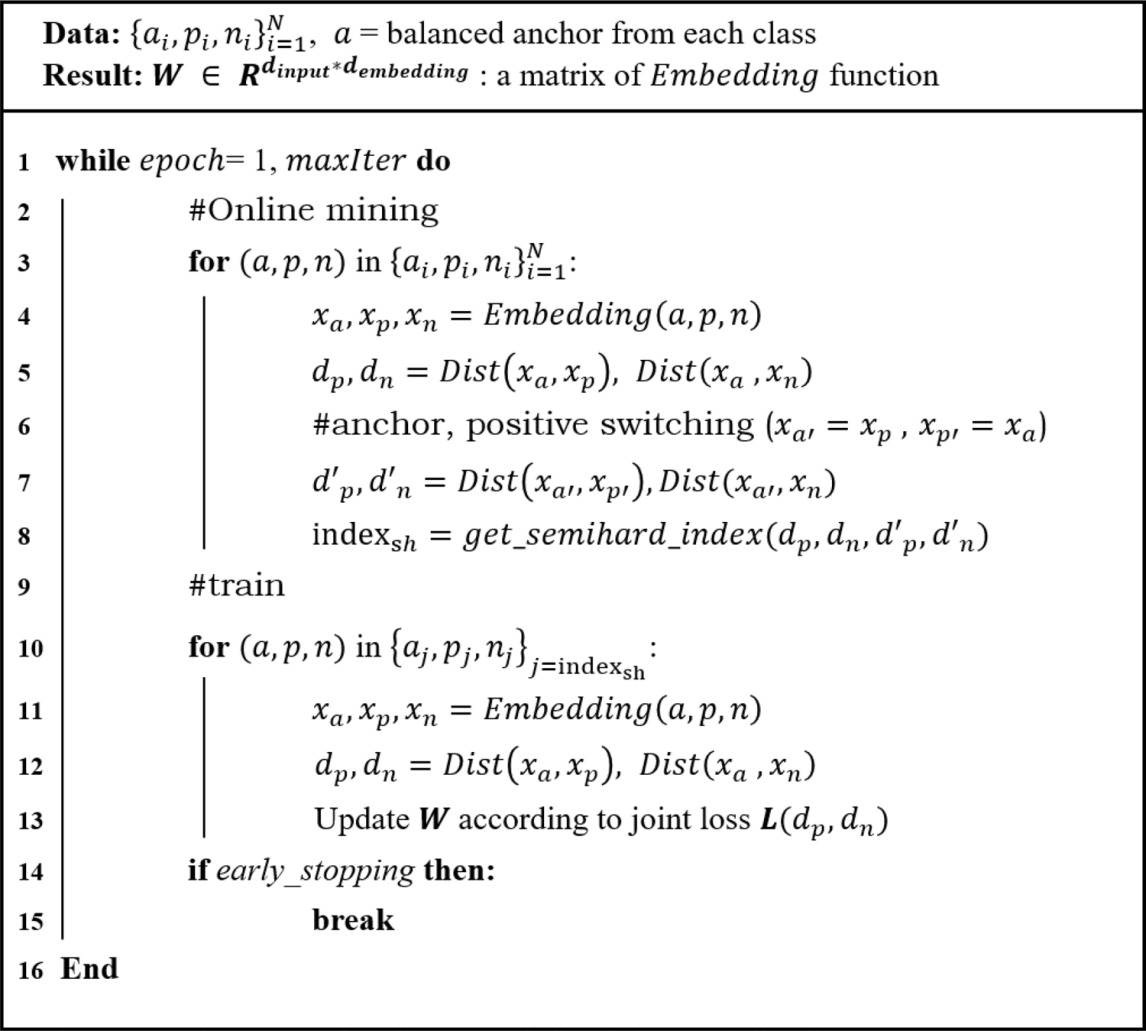
Pseudocode of the proposed JTSC algorithm.

As previously mentioned, the semi-hard sample is defined according to $d_p$ and $d_n$. However, triplet anchors play an important role in defining the category of triplet samples. The category of triplet data can be changed depending on which one is an anchor and which one is a positive sample. For instance, a semi-hard sample could change to a hard sample in certain cases when switching from an anchor to positive, and positive to an anchor (Fig.  2). Therefore, such triplets are uncertain semi-hard samples because the categories change according to the anchor selection in the two samples from the same class in the triplet data structure. To address the uncertainty of these semi-hard samples, we additionally consider that the semi-hard condition is still satisfied in the switching condition. Therefore, we double-checked all semi-hard samples among the constructed triplets during the training of the embedding function. These strict semi-hard samples were updated in every epoch by online mining. The pseudocode for updating the weights of the embedding function is given in Fig. 3.

Moreover, we added the loss term $l_{switching}$ to the switched anchor $x_{a'} (=x_p)$ and switched positive $x_{p'} (=x_a)$ samples to weigh semi-hard samples in the anchor-positive switching condition. Therefore, we selected strict semi-hard samples using the proposed triplet mining method and weighted them with switching loss. The loss term of JTSC used in training is defined as follows:

$$\begin{aligned}{} & {} l_{switching} = [||x_{a'}-x_{p'}||^2 -||x_{a'}-x_n||^2 +m]_+,\\{} & {} l_{joint} = l_{triplet} + l_{switching} + l_{angular} \\{} & {} l_{joint} = [||x_a-x_p||^2 - ||x_a-x_n||^2 + m]_+ +[||x_{a'}-x_{p'}||^2 -||x_{a'}-x_n||^2 +m]_+ + [||x_a-x_p||^2-4tan^2\alpha ||x_n-x_c||^2]_+ \end{aligned}$$

## Results and discussion

### Training setup for AD data

The 1D-CNN model consists of a 1-dimensional convolutional layer, a max pooling layer, and a fully connected layer to embed in the vector space used for deep metric learning. We adopted the learning rate $\in [0.1,0.05,0.01,0.005]$ for training and performed the Xavier initialization for the weights of the 1D-CNN. The size of the embedding vector $\in$ [30,60,90,120,150] was set as a hyperparameter, which is the vector size for representing the gene expression data through the 1D-CNN model. The degree $\alpha \in [30,45,60,75]$ of the angular loss was also used as a hyperparameter. The hyperparameters were determined using the validation dataset. The sizes of kernels in the 1D-CNN model were set to 200, 150, and 200, which were about 1% of the number of genes in ANM1, ANM2, and ADNI, respectively, similar to in a previous study^30^. The model sets the stride size of the kernel equal to the kernel size in order to extract important features from non-overlapping genes. The training batch size was set to 300. We then constructed 50,000 triplets of three data points: anchor, positive, and negative samples for ANM1 and ANM2. For training with a balanced number of anchors, data consisted of 25,000 triplets of random AD anchors and 25,000 triplets of random control anchors. However, considering the low performance of the baseline from other machine learning methods on the ADNI dataset, we constructed a larger dataset with 100,000 triplets, including 50,000 AD anchors and 50,000 control anchors. For each anchor in two classes, the positive sample was extracted from the same label as the anchor, and the negative sample was extracted from the opposite label of the anchor. We loaded all triplets that we constructed and calculated the Euclidean distance between the output vectors of three data points (anchor, positive, and negative) through the 1D-CNN model. Owing to the anchor dependency in the triplet category definition, we selected strict semi-hard samples for training the 1D-CNN model, additionally considering the semi-hard constraint. The Adam optimizer with mini-batch was employed in the training step. Subsequently, the joint loss was used as a combination of triplet loss, angular loss, and switching loss for training. After embedding the training, validation, and test sets, a fully connected network was added to classify the disease and controls using these features. The fully connected one-layer neural network was initialized with Xavier initialization and was trained using the Adam optimizer. All experiments were performed at a margin *m* (> 0) of 0.5, where a margin indicates the degree to which $d_n$ is judged to be close to $d_p$ and determines the number of semi-hard samples from the easy samples during training.

### Evaluation in AD data

For JTSC, hard and semi-hard samples whose triplet categories changed when switching between the anchor and positive samples were filtered out. Strict semi-hard samples that satisfied the semi-hard constraint were used for training. Thus, training the embedding functions with these semi-hard samples could converge more quickly than training with all possible triplets. We calculated the area under the curve (AUC), area under the precision-recall curve (AUPRC), and F1 score to measure the performance of the AD prediction model using ten-fold cross-validation.

The performance of the proposed method was compared with simple machine learning-based models: support vector machine (SVM), random forest (RF), XGBoost (XGB), single layer neural network (NN), 1D-CNN, and other augmentation methods using GAN: SVM with GAN and 1D-CNN with GAN. These machine learning methods were implemented with the sklearn and xgboost python packages. For the SVM with GAN, GAN was applied to the data augmentation using a one-layer neural network for both the generator and discriminator. The input was embedded into the embedding vector with a dimension of 512 and a latent variable was randomly extracted from a normal distribution. We generated the same number of augmented data points as that of training data in each fold through the conditional GAN model^38^. Then, an SVM classifier was trained using both the training and generated data.

When measuring the prediction performance with AD gene expression data (Table 1), the RF model showed the lowest performances for ANM1 and ADNI. The performances of both SVM and XGB were lower than that of 1D-CNN with clinical embedding, except for ANM2. Performance improvements were observed in SVM with GAN when compared with SVM without data augmentation on the AD dataset. Similarly, when we applied GAN augmentation to 1D-CNN, the performances were improved compared to 1D-CNN except for AUC values in ADNI. Furthermore, 1D-CNN with clinical embedding model performed better than using GAN, confirming that clinical information was effectively concatenated. In ANM2, XGB, a decision tree-based model, showed the highest performance of AUPRC, but 1D-CNN with clinical embedding model outperformed the other baseline methods. However, our JTSC model outperformed all other models with AUC values of 0.887, 0.765, and 0.652 for ANM1, ANM2, and ADNI, respectively, and consistently showed best performances for AUPRC and F1, except for AUPRC in ANM2, as shown in Table 1. These results reveal that classifiers with vector representations from deep metric learning result in more accurate prediction models than other machine learning methods.

**Table 1:** Performance of Alzheimer’s disease (AD) classification.

Models	ANM1			ANM2			ADNI		
	AUC	AUPRC	F1	AUC	AUPRC	F1	AUC	AUPRC	F1
RF	0.697	0.697	0.543	0.683	0.634	0.67	0.512	0.349	0.481
SVM	0.775	0.795	0.703	0.676	0.626	0.668	0.584	0.393	0.569
SVM with GAN	0.811	0.798	0.703	0.688	0.627	0.67	0.589	0.398	0.579
XGB	0.856	0.827	0.802	0.743	**0.802**	0.738	0.565	0.461	0.616
NN	0.788	0.728	0.641	0.723	0.754	0.713	0.534	0.358	0.47
1D-CNN	0.827	0.825	0.81	0.725	0.739	0.726	0.624	0.474	0.626
1D-CNN with GAN	0.861	0.891	0.798	0.728	0.749	0.735	0.608	0.486	0.628
1D-CNN with clinical embedding	0.858	0.887	0.802	0.743	0.755	0.744	0.631	0.495	0.658
JTSC	**0.887**	**0.906**	**0.83**	**0.765**	0.776	**0.767**	**0.652**	**0.536**	**0.678**

The highest values are in bold.

**Figure 4:**
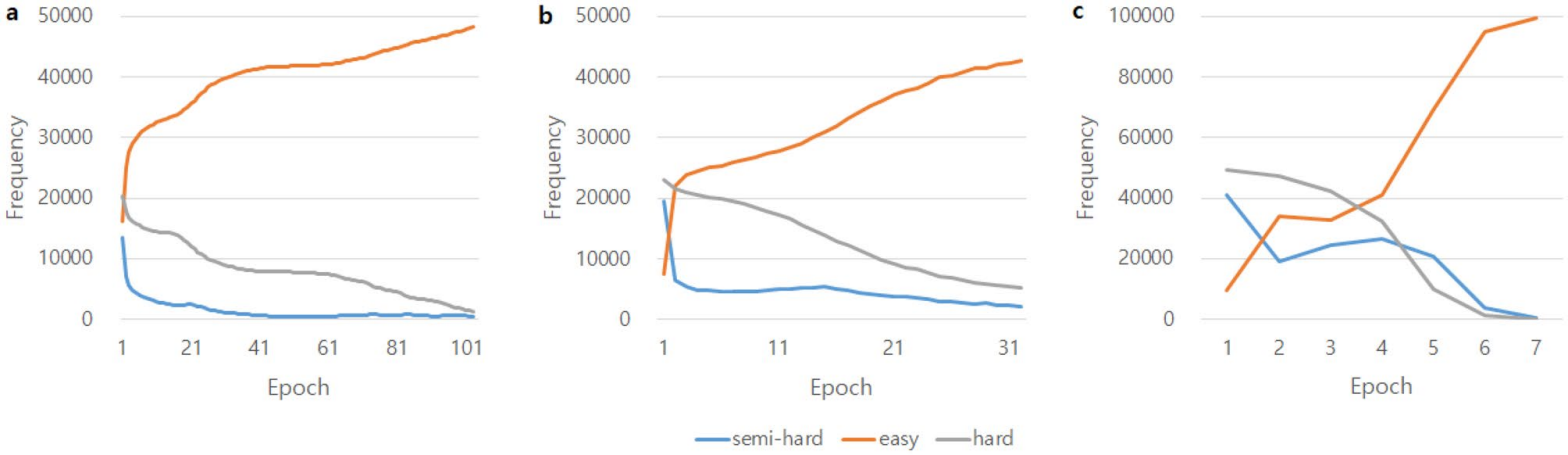
Changes in the distribution of each triplet category during the training in (**a**) ANM1, (**b**) ANM2, and (**c**) ADNI.

In JTSC, even when training with large triplet data, the model quickly arrived at an optimized model with strict semi-hard mining. During the training epoch, we counted the number of triplets in the easy, semi-hard, and hard categories to verify whether vector embedding through an embedding function was appropriately trained. Figure 4 shows for the ANM1, ANM2, and ADNI datasets that the number of easy samples increased while the number of semi-hard and hard samples decreased as the learning progressed, as hard and semi-hard samples were converted into easy samples. The training was terminated to prevent overfitting via early stopping when the validation loss did not decrease after 30 epochs, or when the number of semi-hard samples to train was less than 50.

In addition, when hard samples were included in the training, learning ended before a sufficient number of easy samples were generated, and an oscillation interval occurred (Supplementary Fig. S1). The training was also affected by the number of hard samples used. The training process with semi-hard mining containing 500 hard samples in the order of least loss among the hard samples showed a smoother learning pattern than that containing full hard samples. The AUC also decreased when additional hard triplet data were used (Supplementary Table S4).

### Ablation study

An ablation study was conducted to demonstrate the impact of components of JTSC (Table 2). All experiments were performed using semi-hard mining with online learning at every epoch. Initially, the performance changes between the conventional semi-hard mining (triplet loss and angular loss) and the addition of a constraint to the extracted semi-hard samples were checked. In most cases, we noticed a marked improvement in AUC values by imposing a constraint on the semi-hard samples. One exception is when adding semi-hard constraint to the triplet loss in ADNI. A slight reduction in performance from 0.611 to 0.593 was observed. Subsequently, the contribution of each of loss (angular loss and switching loss) compared to the joint loss of JTSC was checked. Employing a single loss with the constraint model resulted in a lower prediction performance than the joint loss of JTSC. Nonetheless, the extent of contribution to performance varied slightly depending on the dataset. Specifically, in ANM1, a comparable level of performance contribution was observed between the angular loss with constraint model and the switching loss with constraint model. In ANM2, the switching loss ($l_{switching}$) exerted a more substantial influence on prediction performance compared to $l_{angular}$. Conversely, in the ADNI dataset, $l_{angular}$ exhibited the most pronounced effect on prediction performance. Notably, the triplet loss with the constraint model demonstrated the lowest prediction performance in ANM1 and ADNI under stringent semi-hard mining conditions. Furthermore, the triplet loss with constraint model was similar or lower than the performances of the 1D-CNN with clinical embedding in Table 1. The JTSC model that combined triplet loss, angular loss and switching loss accurately selected semi-hard samples, and showed the best performance in AD prediction. These findings underscore that, even when learning from the same set of semi-hard samples under a semi-hard constraint, the information derived from the combination loss significantly influences the training of the embedding function. The combination of a strict semi-hard mining method and joint loss in JTSC proves to be highly effective for representing input gene expression data.

**Table 2:** Ablation study of JTSC.

Models for ablation study	Semi-hard constraint	${l_{triplet}}$	${l_{angular}}$	${ l_{switching}}$	ANM1	ANM2	ADNI
Triplet loss		✓			0.758 ± 0.049	0.715 ± 0.039	0.611 ±0.055
Angular loss		✓	✓		0.786 ± 0.033	0.732 ± 0.025	0.52 ± 0.082
Triplet loss + constraint	✓	✓			0.861 ± 0.011	0.743 ± 0.03	0.593 ± 0.047
Angular loss + constraint	✓	✓	✓		0.874 ± 0.022	0.739 ± 0.037	0.639 ± 0.044
Switching loss + constraint	✓	✓		✓	0.872 ± 0.025	0.746 ± 0.031	0.624 ± 0.041
JTSC + ordered by pathway	✓	✓	✓	✓	0.889 ± 0.018	0.761 ± 0.019	0.654 ± 0.039
JTSC + cosine similarity	✓	✓	✓	✓	0.867 ± 0.027	0.745 ± 0.023	0.605 ± 0.029
JTSC	✓	✓	✓	✓	0.887 ± 0.019	0.765 ± 0.022	0.652 ± 0.037

^#^Performances are shown in AUC values with standard deviations.

In our JTSC approach, input genes were randomly ordered in 1D-CNN. Nevertheless, to explore the potential benefits of incorporating gene locality information, an assessment was conducted by arranging input genes based on pathway information. Gene ontology (GO) biological process terms were used to group genes into pathways. Genes within the same pathways were positioned as neighboring elements in 1D-CNN. Note that genes appearing in multiple pathways were handled without duplication. Table 2 shows that performances using pathway information (JTSC + ordered by pathway) were within standard deviations of the JTSC performances, suggesting that gene order did not play a significant role in JTSC. In a typical CNN model, locality plays a crucial role. However, in this study, the order of the features seemed to have little impact on performance. This can be attributed to the fact that a relatively large (1 %) of all features were computed within a single kernel.

Furthermore, in the ablation study, we opted for cosine similarity to compute the loss instead of Euclidean distance. Unlike Euclidean distance, distance from cosine similarity computes relative comparisons. However, in JTSC using cosine similarity, performance degradation was observed as shown in Table 2.

### Evaluation in TCGA data

To evaluate our method in another task, an experiment was performed to classify early- and late-stage cancers using their gene expression profiles. To train the embedding function through 1D-CNN, 30,000 triplets were constructed for the training dataset and 10,000 triplets for the validation dataset, and the number of anchors in the early and late stages was set to be a half the number of the triplet. Clinical information, such as age and sex, was concatenated for the experiment, and specifically included in every kernel calculation in the 1D-CNN model. Five-fold cross-validation was performed with Xavier initialization. Hyperparameters, such as embedding feature dimension, patience for early stopping, and degree of angular constraint, were selected using the validation set. Then, the performance of JTSC was compared with SVM, RF, XGB, NN and other recent cancer stage classification methods; we also used an original dataset with selected features only (FS)^28^, GAN5 (G5)^28^ and multiple kernel learning (MKL)^27^, as shown in Table 3. To select important features from genes, Kwon et al.^28^ additionally used DNA mutation data from the GDC Data Portal. Common protein-coding genes between the mutation and gene expression data were selected, which resulted in 16,804 common genes in STAD and 16,391 common genes in the remaining cancer type data. In each fold, the RandomForestClassifier from scikit-learn was performed in Python and genes with higher feature importance were selected using all the parameters described in the paper. A generator model G, which consisted of a single layer encoder and decoder and discriminator with two layers, generated multiples of the training data with selected features from FS^28^. From the training set, G5 generated five times the number of samples for gene expression data, which generally performed well in the paper^28^. Then, SVM was used as a classifier. MKL is an SVM-based method for finding the weighted combination of kernels by solving the inner optimization problem^27^. MKL was performed and a validation set was used to search for parameters that optimize the weight of the kernel.

**Table 3:** Performance of early- and late-stage cancer classification.

AUC	BRCA	COAD	ESCA	HNSC	KICH	KIRC	LIHC	LUAD	LUSC	PAAD	READ	STAD	TGCT	THCA
RF	0.501	0.5	0.525	0.5	0.605	0.706	0.589	0.587	0.595	0.605	0.5	0.505	0.678	0.666
XGB	0.577	0.473	0.627	0.633	0.617	0.766	0.667	0.63	0.622	0.582	0.508	0.648	0.721	0.634
NN	0.555	0.492	0.549	0.698	0.694	0.768	0.651	0.597	0.557	0.62	0.571	0.608	0.634	0.68
1D-CNN	0.47	0.466	0.673	0.603	0.678	0.737	0.631	0.549	0.563	0.62	0.415	0.609	0.622	0.568
1D-CNN with clinical embedding	0.476	0.467	0.709	0.666	0.704	0.733	0.669	0.621	0.629	0.656	0.571	0.626	0.665	0.854
SVM	0.542	0.506	0.547	0.516	0.686	0.717	0.619	0.593	0.589	0.554	0.493	0.555	0.646	0.666
SVM with FS	0.5	0.494	0.549	0.511	0.595	0.632	0.63	0.576	0.592	0.563	0.527	0.632	0.618	0.787
SVM with G5	0.515	0.56	0.56	0.523	0.607	0.646	0.594	0.573	0.588	0.573	0.5	0.618	0.634	0.755
MKL	**0.612**	0.504	0.613	0.63	0.694	0.77	**0.719**	**0.658**	**0.732**	0.483	**0.812**	0.623	0.7	0.764
JTSC	0.606	**0.65**	**0.763**	**0.746**	**0.752**	**0.775**	0.675	0.618	0.631	**0.67**	0.601	**0.665**	**0.737**	**0.888**

The highest values are in bold.

The predictive performance for nine cancer types, COAD, ESCA, HNSC, KICH, KIRC, PAAD, STAD, TGCT, and THCA, was the best in JTSC. In particular, for cancer types with less than 100 samples in a class (e.g., COAD, ESCA, HNSC, KICH, READ, STAD, TGCT), JTSC performed the best, with the exception of READ (Supplementary Table S2). However, five of the remaining cancers performed better on MKL. In addition, compared to SVM that used the entire gene, the prediction performance of FS declined for half of the cancer types. The results of the G5 model trained on the generated data of the selected features exhibited performance that was better or similar to FS, but still had a performance lower than JTSC. Note that the MKL method used the expression values of specific genes in pathways, which incorporated the pathway knowledge. Although JTSC did not use the pathway knowledge, JTSC performed better than MKL in 9 out of 14 cancer types.

## Conclusion

In the present study, a novel strict semi-hard mining method with constraint and joint triplet loss for deep metric learning is proposed. In distance metric learning, for tasks that train with a large number of data points, such as image classification, hard mining is more efficient than using whole triple training data^39^. However, for tasks with a small number of samples and a large number of features, such as gene expression data, the semi-hard mining method can improve the prediction performance. To obtain a model with better performance, the semi-hard samples that were converted to hard or easy samples when the anchor labels and positive labels were exchanged were removed. Unlike other triplet mining methods that use all triplet data for training, the embedding function was trained in a more sophisticated manner by considering the distance between embedded samples in certain conditions. In addition, angular loss was added to alleviate the scale dependence of a triplet loss, along with a distance-based objective function in the training. As a result, the sampling method and joint loss improved prediction performance on AD and TCGA datasets.

### Supplementary Information

Supplementary Information.

## Data Availability

We provide the code of JTSC and the splits used for sample dataset (https://github.com/DMCB-GIST/JTSC).
