# Supplementary material for: Joint triplet loss with semi-hard constraint for data augmentation and disease prediction using gene expression data
Source: Sci Rep. 2023 Oct 24;13:18178. doi: 10.1038/s41598-023-45467-8 (PMC10598120; doi:10.1038/s41598-023-45467-8)
Supplement: Supplementary file 1 — Supplementary Information. [file 41598_2023_45467_MOESM1_ESM.docx]

**Supplementary Materials**

**Joint triplet loss with semi-hard constraint for data augmentation and disease prediction using gene expression data**

Yeonwoo Chung^1^ and Hyunju Lee^1,2,*^

^1^School of Electrical Engineering and Computer Science, Gwangju Institute of Science and Technology, Gwangju, 61005, Republic of Korea

^2^Artificial Intelligence Graduate School, Gwangju Institute of Science and Technology, Gwangju 61005, Republic of Korea

*hyunjulee@gist.ac.kr

**Table S1. Characteristics of participants in AD dataset**

|  | ANM1 | | ANM2 | | ADNI | |
| --- | --- | --- | --- | --- | --- | --- |
|  | AD | Con | AD | Con | AD | Con |
| N | 145 | 104 | 140 | 135 | 116 | 246 |
| Age (years, mean ± SD) | 75.4 ± 6.58 | 72.3 ± 6.33 | 77.9 ± 6.74 | 75.3 ± 6.08 | 77.3 ± 7.67 | 76.2 ± 6.49 |
| Gender (men, %) | 46 (31%) | 42 (40%) | 55 (39%) | 54 (40%) | 75 (64%) | 117 (47%) |
| Education (years, mean ± SD) |  | | | | 15.9 ± 2.91 | 16.4 ± 2.68 |
| Marriage (score, mean ± SD) |  |  |  |  | 1.7 ± 0.66 | 1.4 ± 1.14 |

**ANM** AddNeuroMed, **ADNI** Alzheimer’s Disease Neuroimaging Initiative, **AD** Alzheimer’s disease, **Con** control, **SD** standard deviation

**Table S2. Summary of 14 TCGA cohorts used in experiments**

| Cohort | Disease | Early | Late | Total |
| --- | --- | --- | --- | --- |
| BRCA | Breast Invasive Carcinoma | 182 | 913 | 1095 |
| COAD | Colon Adenocarcinoma | 45 | 239 | 284 |
| ESCA | Esophageal Carcinoma | 18 | 166 | 184 |
| HNSC | Head and Neck Squamous Cell Carcinoma | 27 | 492 | 519 |
| KICH | Kidney Chromophobe | 21 | 45 | 66 |
| KIRC | Kideny Renal Clear Cell Carcinoma | 268 | 266 | 534 |
| LIHC | Liver Hepatocellular Carcinoma | 172 | 200 | 372 |
| LUAD | Lung Adenocarcinoma | 266 | 232 | 498 |
| LUSC | Lung Squamous Cell Carcinoma | 240 | 252 | 492 |
| PAAD | Pancreatic Adenocarcinoma | 21 | 157 | 178 |
| READ | Rectum Adenocarcinoma | 12 | 81 | 93 |
| STAD | Stomach Adenocarcinoma | 50 | 325 | 375 |
| TGCT | Testicular Germ Cell Tumors | 56 | 83 | 139 |
| THCA | Thyroid Carcinoma | 284 | 221 | 505 |

**Early** early-stage, **Late** late-stage

**Table S3. Characteristics of participants in TCGA dataset**

|  | N | Age (years, mean ± SD) | Gender (men, %) |
| --- | --- | --- | --- |
| BRCA Early  Late | 182  913 | 59.85 ± 13.21  58.1 ± 13.1 | 0 (0%)  12 (1%) |
| COAD Early  Late | 45  239 | 66.2 ± 12.51  64.7 ± 13.41 | 26 (57%)  130 (54%) |
| ESCA Early  Late | 18  166 | 67.7 ± 11.71  61.8 ± 11.83 | 11 (61%)  147 (88%) |
| HNSC Early  Late | 27  492 | 61.8 ± 15.5  60.8 ± 11.65 | 14 (51%)  369 (75%) |
| KICH Early  Late | 21  45 | 48.6 ± 14.77  52.8 ± 14.03 | 11 (52%)  28 (62%) |
| KIRC Early  Late | 266  268 | 59.7 ± 12.62  61.4 ± 11.57 | 162 (60%)  184 (69%) |
| LIHC Early  Late | 172  200 | 60.5 ± 12.19  58.5 ± 14.46 | 122 (70%)  129 (64%) |
| LUAD Early  Late | 266  232 | 65.9 ± 9.92  64.7 ± 9.89 | 110 (41%)  121 (52%) |
| LUSC Early  Late | 240  252 | 68.2 ± 8.13  66.1 ± 8.88 | 172 (71%)  192 (76%) |
| PAAD Early  Late | 21  157 | 65.5 ± 12.13  64.4 ± 10.8 | 10 (47%)  88 (56%) |
| READ Early  Late | 12  81 | 64 ± 13.47  62.7 ± 12.36 | 8 (66%)  44 (53%) |
| STAD Early  Late | 50  325 | 68.6 ± 10.95  64.5 ± 10.54 | 35 (70%)  211 (65%) |
| TGCT Early  Late | 56  83 | 33.7 ± 11  30.5 ± 7.52 | 56 (100%)  83 (100%) |
| THCA Early  Late | 284  221 | 38.2 ± 13.03  58.8 ± 10.83 | 68 (23%)  68 (31%) |

**Early** early-stage, **Late** late-stage, **SD** standard deviation

**Table S4. Classification performance (AUC) with and without hard samples in Alzheimer’s Disease dataset**

|  | ANM1 | ANM2 | ADNI |
| --- | --- | --- | --- |
| Proposed (without hard samples) | 0.887 | 0.765 | 0.652 |
| Proposed method with hard samples | 0.819 | 0.749 | 0.622 |

**Figure S1. Distribution of triplet category in Alzheimer’s Disease dataset.** (a) semi-hard mining, (b) semi-hard mining with 500 hard samples, (c) semi-hard mining with whole hard samples. Changes in the number of triplet categories oscillated differently with the number of hard samples. When 500 samples with the smallest loss among hard samples were selected and trained together, the variance was larger compared to semi-hard mining. The proposed method with all hard samples had the most frequent oscillations. The embedding function seemed to be well trained with hard samples in ANM1, but it showed a noticeable performance drop in 10-fold cross-validation for measuring the prediction performance.

1. ANM1

1. ANM2

1. ADNI
